# Numbers, graphs and words – do we really understand the lab test results accessible via the patient portals?

**DOI:** 10.1186/s13584-020-00415-z

**Published:** 2020-10-28

**Authors:** Shirly Bar-Lev, Dizza Beimel

**Affiliations:** 1 Dror (Imri) Aloni Center for Health Informatics, Tel Aviv, Israel; 2 Department of Industrial Engineering and Management, Ruppin Academic Center, Emek Hefer, Israel; 3 Department of Computer and Information Sciences, Ruppin Academic Center, Emek Hefer, Israel

**Keywords:** Health information technology, Information format, Patient engagement, Care seeking

## Abstract

**Background:**

The heavy reliance on remote patient care (RPC) during the COVID-19 health crisis may have expedited the emergence of digital health tools that can contribute to safely and effectively moving the locus of care from the hospital to the community. Understanding how laypersons interpret the personal health information accessible to them via electronic patient records (EPRs) is crucial to healthcare planning and the design of services. Yet we still know little about how the format in which personal medical information is presented in the EPR (numerically, verbally, or graphically) affects individuals’ understanding of the information, their assessment of its gravity, and the course of action they choose in response.

**Methods:**

We employed an online questionnaire to assess respondents’ reactions to 10 medical decision-making scenarios, where the same information was presented using different formats. In each scenario, respondents were presented with real (anonymized) patient lab results using either numeric expressions, graphs, or verbal expressions. Participants were asked to assess the gravity of the hypothetical patient’s condition and the course of action they would follow if they were that patient. The questionnaire was distributed to more than 300 participants, of whom 225 submitted usable responses.

**Results:**

Laypersons were more likely to overestimate the gravity of the information when it was presented either numerically or graphically compared to the narrative format. High perceived gravity was most likely to produce an inclination to actively seek medical attention, even when unwarranted. “Don’t know” responses were most likely to produce an inclination to either search the Internet or wait for the doctor to call.

**Policy recommendations:**

We discuss the study’s implications for the effective design of lab results in the patient portals. We suggest (1) that graphs, tables, and charts would be easier to interpret if coupled with a brief verbal explanation; (2) that highlighting an overall level of urgency may be more helpful than indicating a diversion from the norm; and (3) that statements of results should include the type of follow-up required.

## Introduction

The heavy reliance on remote patient care (RPC) during the COVID-19 health crisis may have expedited the use of digital health tools to safely and effectively move the locus of care from the hospital to the community, and even the home [1, 2]. Enthusiasts see this as a long-overdue opportunity to reengineer care processes so as to reap the full benefits of health information technologies [3]. Health information technologies (HIT) are broadly defined as “the electronic systems that health care providers and increasingly, patients, use to store, share and analyze information” [4]. Direct patient use of test data is consistent with trends toward patient-centered care and the medical home concept, which aims to achieve greater patient involvement in both medical decision making and health self-management [5]. These care approaches expect the “digitally engaged patient” to self-monitor and self-care for themselves and their families through the skillful use of enhanced digital technologies [6] (see also [7–11]). Yet managing their own health has been shown to place heavy demands on laypersons, who are now expected to correctly interpret their test results, evaluate the pros and cons of different treatments, and decide on a preferred course of action [12, 13]. Correspondingly, clinicians have expressed concern that patients often experience great difficulty in comprehending, interpreting, and correctly responding to personalized health information, partly due to inappropriate presentation of the information in patient portals [13, 14]. In particular, misunderstanding test results leads to confusion, frustration, and disruptions in healthcare processes, including delays in seeking care, overutilization of services, medication errors, and inappropriate healthcare decision-making [5, 7, 14].

A stream of evidence shows that laypersons differ from experts in how they assess the meaning of healthcare information and evaluate its trustworthiness [15–18]. For instance, in the case of antenatal screening tests, practitioners tend to frame risk information numerically, as the probability of a genetic condition, even though laypersons display a better understanding of the information when a verbal or narrative format is used [19]. Yet patients are more likely to take up genetic testing when presented with numeric risk information [18, 20, 21]. Compared with medical professionals, laypersons are more readily influenced by the attractiveness of a site’s design and have even been found to reject high-quality content because of poor visual design, confusing displays, and a low density of relevant information [6, 16]. Since laypersons can and often do view the results of their check-ups and medical tests prior to interacting with their healthcare providers, the meaning they attach to these results can significantly affect their decision-making and subsequent follow-up care.

Recent definitions of e-health literacy consider the set of individual capacities that allow the person to acquire and use new information, as well as the cognitive competencies required to make judgments and decisions in everyday life concerning health [22, 23]. E-health literacy has been shown to combine knowledge and skills from a wide variety of domains, and is inherently contingent upon the social contexts wherein it is developed and expected to be put into use. As a result, it can be affected by a variety of personal and socio-demographic factors, including age, gender, education, acute and chronic health concerns, general health literacy, and technological proficiency [24, 25]. However, it is still difficult to assess whether people’s interpretation of information such as test results is more affected by these socio-demographic variables, or by how the information is displayed. Thus, the first aim of the present study is to help address this gap by examining respondents’ interpretation of medical information while controlling for various demographic characteristics (age, education level, socio-demographic status, health status, attitudes towards self-care, and responsiveness to medical or health recommendations).

The present research is also motivated by the fact that despite growing interest in laypersons’ comprehension and use of medical knowledge to reach appropriate medical decisions, little is still known of the extent to which different visual displays help people discriminate between test results that do or do not require urgent action [7, 13, 14, 26]. Zikmund-Fisher et al. [13] asked their participants to imagine that they were viewing the results of a set of blood tests on an online patient portal. They varied the format in which participants viewed these test results – as a number line graph, a table, or raw numbers – and tested the relationship between the display format and respondents’ perceptions of urgency and inclination to contact health care providers. They found that when test results were abnormal (indicated extreme values), perceived urgency was universally high, regardless of which display format was shown. However, perceptions regarding near-normal values varied substantially across formats. The pattern they found was consistent: participants who saw their near-normal values in a tabular display rated those results as most urgent, while those who saw a gradient line display perceived the results as least urgent. The authors attributed these differences in interpretation to both the amount of information conveyed by each format, and to the cognitive skills each format requires to deduce the bottom-line implications of the information. Thus, the second aim of the present study is to test whether the findings of Zikmund-Fisher et al. [13] are conceptually replicated in a different sample and study design.

Following Zikmund-Fisher et al. [13], we examine three aspects of knowing related to the interpretation of lab test results in patient health portals: knowing, uncertainty, and accuracy. Knowing is defined here according to the standard dictionary definition, as a perception of clear and certain mental apprehension. Uncertainty is defined as a cognitive claim of insufficient knowledge, or a lack of understanding regarding the meaning of the information presented [27, 28]. Accuracy is defined as a condition or quality of being correct or exact with relation to a standard, again based on the standard dictionary definition. More concretely, we examine how different displays of information were related to different interpretations of severity, “do not know” answers and inaccuracies in judgment.

We propose three hypotheses:

- H1: The three information formats (verbal, numeric, and graphic) will differ in the accuracy of participants’ assessments.
- H2: The three information formats (verbal, numeric, and graphic) will yield different levels of uncertainty with regard to the condition’s level of gravity.
- H3: The higher the perceived gravity of the health condition, the more proactive people are likely to be in seeking help or information.

**Fig. 1:**
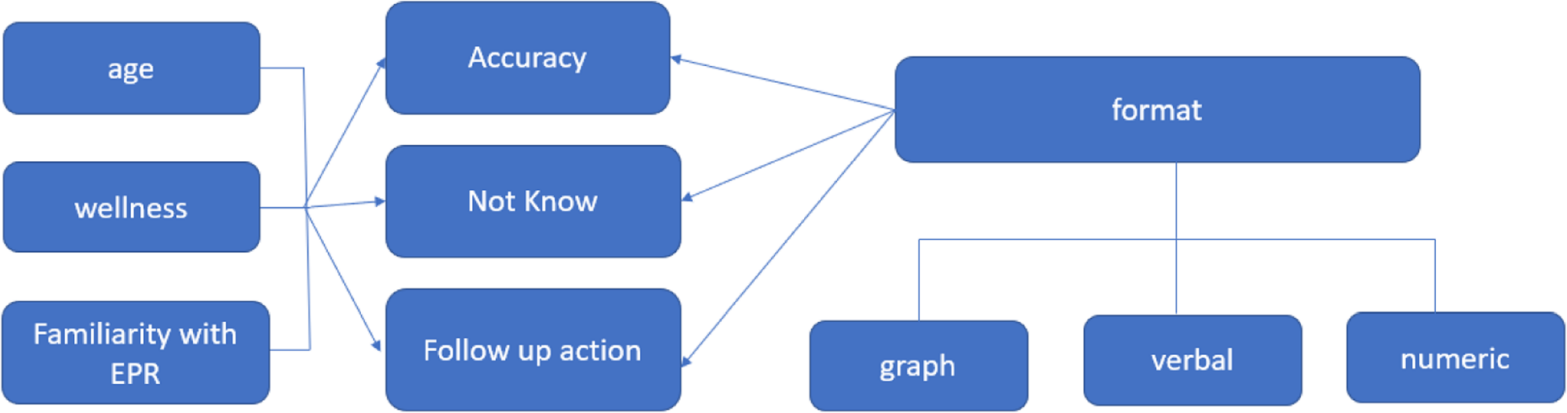
Conceptual model

The conceptual model which forms the basis for the hypotheses is displayed in Fig. 1.

## Methods

### Design and sample

We employed a survey to assess respondents’ reactions to 10 medical decision-making scenarios, where the same information was presented using different formats. In each scenario, respondents were presented with real (anonymized) patient lab results using either numeric expressions, graphs, or verbal expressions. Participants were asked to assess the gravity of the hypothetical patient’s condition and the course of action they would follow if they were that patient.

**Fig. 2:**
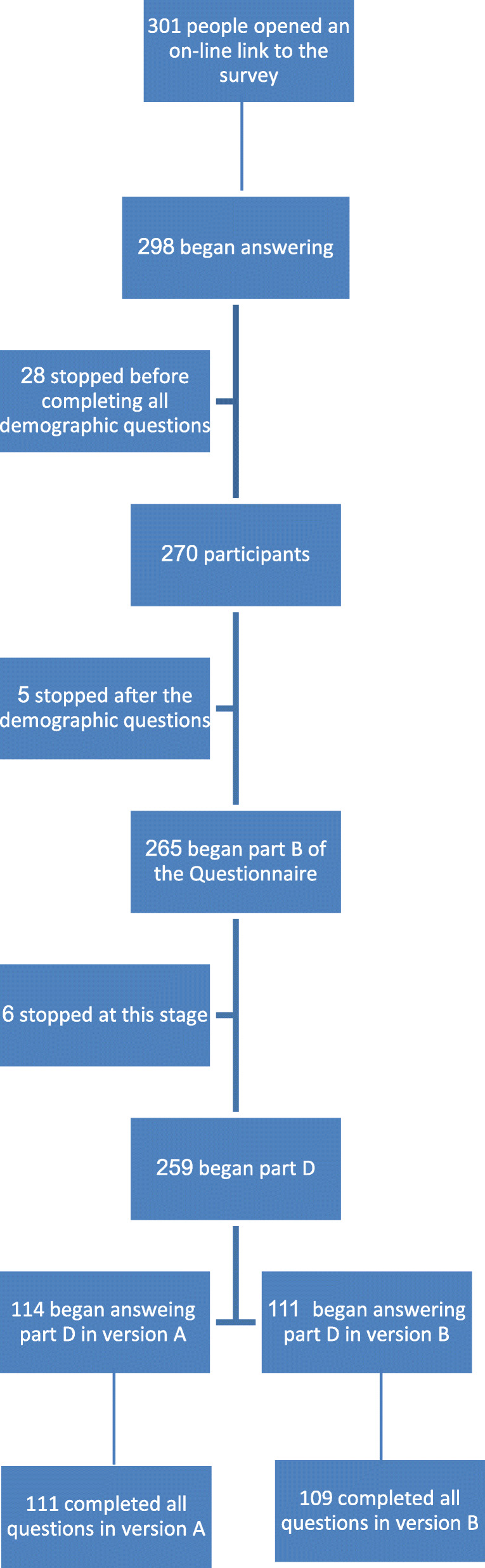
Data flow chart displaying treatment of missing values

Participants were recruited through convenience sampling, mainly via the authors’ networks. This sampling method is commonly employed in healthcare-related surveys [29–31]. Each respondent was asked to virally distribute the link to others in their network (snowball sampling). The link was operative for a period of 2 weeks, and we monitored the response rate daily. By the end of the data collection period the link had been distributed to over 300 individuals. We used all valid responses obtained. That is, rather than trimming or imputing, we worked with different sample sizes for each analysis, calculating the means for each respondent where necessary. In total, 225 participants returned questionnaires suitable for analysis, meaning they responded to at least some of the scenarios, and 220 returned fully complete questionnaires. Approximately 83% of those who began the survey (i.e., who completed the demographic questions) submitted usable answers – a satisfactory percentage, given that the questionnaire was relatively long and contained 10 different scenarios. Figure 2 describes our missing values policy in detail. Missing data analysis revealed that missing values were random for most variables A Pearson Chi-Square Test showed that those who did not complete the questionnaire differed from those who did in three demographic variables: income (X2 (3, *N* = 270) =15.09 value, *p* = 0.002), and family status (X2 (2, *N* = 270) =18.386 value, *p* < 0.002). An independent t-test revealed that those who did not complete the questionnaire were significantly older (M = 41.87; std. = 12.144) than those who did (M = 35.19, std. = 13.781) F = 2.459, *P* = 0.003). These differences are consistent, meaning that those who did not provide usable responses were significantly older, had slightly higher incomes, and were married. No differences were found in health status. Finally, we applied a binomial test of equal proportions or two-proportion z-test to determine the minimum required sample size. In this study, a random sample of 43 pairs (where the mean difference is 0.22 and the standard deviation of the difference is 0.5) would allow us to declare with 80% power that the mean of the paired differences is significantly different from zero (i.e., a two sided *p*-value is less than 0.05). The sample size of this study is 225, 114 for version A and 109 for version B – comfortably above 43 pairs (see Table 1).

**Table 1:** Descriptive statistics of the sample

	Demographic characteristics of those who completed the whole questionnaire, and those who did not					
	Stopped before Section D		started section D		Total	
	Count	Column N %	Count	Column N %	Count	Column N %
**Total**	**45**	**100%**	**225**	**100%**	**270**	**100%**
sex						
male	13	28.9%	98	44.1%	111	41.6%
female	32	71.1%	124	55.9%	156	58.4%
HMO						
Clalit	19	43.2%	107	47.8%	126	47.0%
Maccabi	18	40.9%	77	34.4%	95	35.4%
Leumit	2	4.5%	18	8.0%	20	7.5%
Meuhedet	5	11.4%	22	9.8%	27	10.1%
Age (mean and Std)	42	Std = 12	M = 35	Std = 14	M = 36	
Income (the national monthly average wage is 7500 nis						
Below average	11	25.0%	121	53.8%	132	49.1%
Average	5	11.4%	17	7.6%	22	8.2%
Above average	23	52.3%	59	26.2%	82	30.5%
Far above average	5	11.4%	28	12.4%	33	12.3%
Education						
High school	7	15.6%	73	32.6%	80	29.7%
Academic	36	80.0%	136	60.7%	172	63.9%
Professional	1	2.2%	2	0.9%	3	1.1%
other	1	2.2%	13	5.8%	14	5.2%
Religiosity						
secular	41	91.1%	196	87.1%	237	87.8%
traditional	2	4.4%	22	9.8%	24	8.9%
observant	2	4.4%	7	3.1%	9	3.3%
Haredi	0	0.0%	0	0.0%	0	0.0%
Familial Status						
Single	11	24.4%	127	57.0%	138	51.5%
Married	34	75.6%	91	40.8%	125	46.6%
Divorced	0	0.0%	0	0.0%	0	0.0%
Widower	0	0.0%	5	2.2%	5	1.9%
Total	45	100.0%	223	100.0%	268	100.0%
Birth Country						
Israel	22	50.0%	126	56.3%	148	55.2%
Former Soviet Union	5	11.4%	23	10.3%	28	10.4%
France	1	2.3%	2	0.9%	3	1.1%
Ethiopia	0	0.0%	1	0.4%	1	0.4%
other	16	36.4%	72	32.1%	88	32.8%
Total	44	100.0%	224	100.0%	268	100.0%

Participants were randomly assigned to receive one of two versions of the questionnaire. Both versions contained the same scenarios and questions, differing only in the presentation of the test results. Of the 225 surveys which contained usable responses, 114 represented the first version of the survey, and 111 the second. For each scenario, two of the three formats were contrasted between the two versions (i.e., sometimes verbal vs. graphic, sometimes graphic vs. numeric, etc.; see under “Procedure and materials” below). An independent-samples t-test showed no differences in demographic measures between respondents who received the two versions of the questionnaire (see Table 2).

**Table 2:** Results of an Independent T-test results for version A and version B of the questionnaire

Variable	Mean	N	Std. Deviation	t-mean
sex				
Version 1	1.51	122	0.501	−1.388
Version 2	1.63	118	0.482	
age				
Version 1	35.50	119	13.807	−0.595
Version 2	35.42	113	14.798	
income				
Version 1	2.02	124	1.121	−0.346
Version 2	1.97	118	1.149	
education				
Version 1	1.81	123	0.646	0.514
Version 2	1.79	119	0.709	
Health status				
Version 1	4.06	124	0.747	0.830
Version 2	4.08	119	0.787	

P = NS

### Procedure and materials

**Fig. 3:**
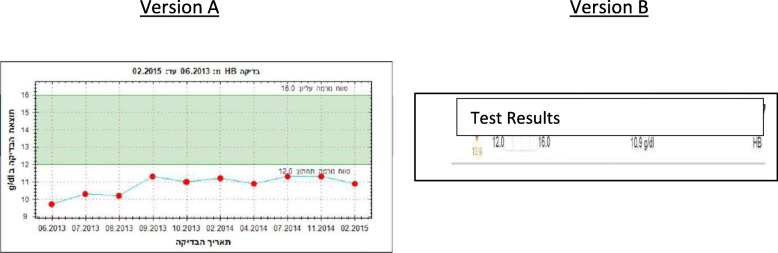
An example of one scenario with graphic (version A) and numeric (version B) presentations of information.For several weeks, Ayala had been feeling more tired than usual. She is pale and experiences a general feeling of laxity. During a visit to her family doctor, he recommended checking her Haemoglobin level. Haemoglobin is a molecule found in red blood cells that carries the oxygen from the lungs to the tissues of the body. Ayala took the test and received the following result

Using Qualtrics software we produced anonymized links to the two versions of the survey, each containing 10 medical decision-making scenarios. The order of the scenarios was the same in both versions. In each scenario, respondents were presented with lab results relating to important but non-life-threatening health conditions using either numeric expressions, graphs, or verbal expressions. Participants were asked to assess the gravity of the hypothetical patient’s condition and the course of action they would follow if they were that patient. The lab tests (blood work or cultures) were extracted from authentic patient portals, and had been ordered to investigate or test for one of the following: erratic menstrual cycles; low hemoglobin; hepatitis B; streptococcus (a throat infection); or a routine cholesterol check. These conditions and tests were chosen because they are relatively common, likely to be only moderately serious, and in most cases potentially applicable to both men and women. Each scenario contained a short description of the patient’s symptoms and the possible consequences of poor treatment. Two physicians, both of them general practitioners working both in the community and hospitals, independently reviewed the scenarios and confirmed the accuracy and reliability of the information provided. An example of one scenario is shown in Fig. 3.

After reading the scenarios and viewing the results, participants were asked to assess the gravity of the condition, and what course of action they would recommend for the patient. Our aim was not to test our participants’ medical proficiency or knowledge, but solely their interpretation of the lab results presented to them. For each scenario, the test results were presented in two of the three different formats (numeric, graphic, or verbal), one in version A and a different one in version B (see Fig. 3). Presentations in the verbal format contained either a diagnosis, or a short explanation and/or recommendation. The numeric format contained a single measure or a series of measures presented in table form, sometimes with an indication of a norm. The graphic format contained a line graph showing the current measurement, previous measurements, and an indication of the norm.

### Measures

Knowing. After reading each scenario, participants were asked to assess the gravity of the hypothetical health condition, based on the results of the lab tests provided. Perceived gravity ranged from 1 = very low to 5 = very high, with 6 = don’t know. Knowing was operationalized as any response from 1 to 5 (as opposed to 6, meaning “don’t know”).

Uncertainty was operationalized as a choice of the sixth option in the gravity scale, namely “don’t know.” A choice of the “don’t know” response indicated that the participant had difficulty interpreting the lab results.

Accuracy, the quality of being correct with respect to a standard, was also measured in relation to responses on the gravity scale, and was operationalized by comparing all responses of 1 to 5 against the physicians’ assessment of the gravity of the health condition. We were thus able to assess who underestimated the gravity of the condition, who correctly assessed the gravity of the condition, and who overestimated the gravity of the condition. This variable ranged from − 0.35 to 3.75. We then turned this into a categorical variable named level of accuracy (> 0 = 1; 0 = 2; < 0 = 3).

#### Preferred course of action

For each scenario, we asked participants to indicate how likely they would be, if they were the patient, to do each of the following upon seeing the presented lab results: 1. immediately contact their doctor; 2. search out more information on the Internet; 3. wait for their physician to contact them; and 4. wait until their next visit to the doctor to verify the meaning of the results. For each course of action, respondents were asked their likelihood of taking that path on a 5-point scale, where 1 = very unlikely and 5 = very likely.

#### Demographics and controls

Demographic measures collected were age, gender, HMO membership, income, education, religiosity, family status, and country of birth (see Table 1). To offset differential response rates by age, we divided the respondents into three distinct age samples (18–39, 40–59, and 60 and older). Income was measured on a three-point scale (“The average income is 7500 NIS [about $2500 a month]. Is your income higher than, equal to, or less than 7500 a month?”). We also controlled for use of EPR systems, respondents’ self-reported health status, attitudes towards self-care, and responsiveness to medical or health recommendations (details on these measures are given under Results below). We carried out reliability analyses on the scales assessing participants’ level of EPR use (8 items) and beliefs about health and healthcare (7 items). Both reached acceptable reliabilities, α = 0.88 and α = 0.620 for the EPR and health beliefs scales, respectively.

## Results

### Descriptive statistics – demographics, health status, health behaviors, and EPR use

Table 1 presents the descriptive statistics of the final sample. As the table shows, the final sample was fairly heterogeneous in its socio-demographic characteristics. With respect to HMO membership, the proportions represented in our sample resemble the proportions in the Israeli population as a whole, with a slight over-representation for one HMO, Maccabi (Clalit = 53.967% vs. 47% in sample; Maccabi = 26.007% vs. 35.4%; Leumit = 7.772% vs. 7.5; Meuhedet = 12.254 vs. 10.1; all data from Social security 2020). As for education, our sample has a relatively high proportion of educated participants. However, it should be noted that 50% of all Israeli citizens aged 25–64 have either tertiary or academic education [32]. Our sample underrepresents Haredi and religious participants, as well as other minority groups. The limitations of the chosen sampling method will be discussed later in the paper.

Based on the full sample, 5% of our respondents claimed to be in poor health, while 88% reported being in good, very good or excellent health. Fifty-five respondents (18%) reported suffering from a chronic illness, and 22 respondents (5%) reported suffering from some type of physical limitation. Being in good or very good health was negatively correlated with age (r [255] = − 0.240, *P* < 0.01). Age was positively correlated with feeling responsible for one’s health (r [213] = 0.139, *P* < 0.001), and negatively correlated with postponing regular checkups (r [213] = − 0.162, *P* < 0.001). Most indicated that they felt responsible for keeping healthy (M = 4.85, std. = 0.468, on a 5-point scale where 1 = strongly disagree, 5 = strongly agree) and that maintaining a healthy lifestyle was important to them (M = 4.69, std. = 0.57; 1 = strongly disagree, 5 = strongly agree). They also reported generally complying with their doctor’s recommended regime (M = 4.36, std. = 0.844). When asked how they respond when they feel sick, 50% of the respondents who answered this question (*N* = 130) said they turn to their doctor for a consultation, while 25% (*N* = 65) turn to a family member, 22.7% (*N* = 59) consult medical websites for information, and only 2.3% (*N* = 6) consult online health forums.

With respect to EPR use, 71% of our participants (*N* = 173) reported that they frequently access their lab results via the EPR. Ten percent (*N* = 30) claimed to have never viewed their lab results via the EPR, and an additional 7.7% (*N* = 23) of our participants were not aware of being able to view their lab results via the EPR. In general, women tend to use the EPR significantly more than men (t [216] = − 3.6, *P* < 0.001). However, these differences disappear when focusing on use of the EPR to view lab results and health recommendations (i.e., women more than men use the EPR for administrative purposes such as scheduling doctors’ appointments and filing requests for prescription drugs for themselves and other family members). There was a significant main effect of age on EPR use, F (2, 246) = 4.718, *P* < 0.000. Participants aged 18–39 were significantly less inclined to consult the EPR than those aged 40–59 and those aged 60+ (*P* < 0.000).

### Hypothesis testing

- H1: The three information formats (verbal, numeric, and graphic) will differ in the accuracy of participants’ assessments.

**Fig. 4:**
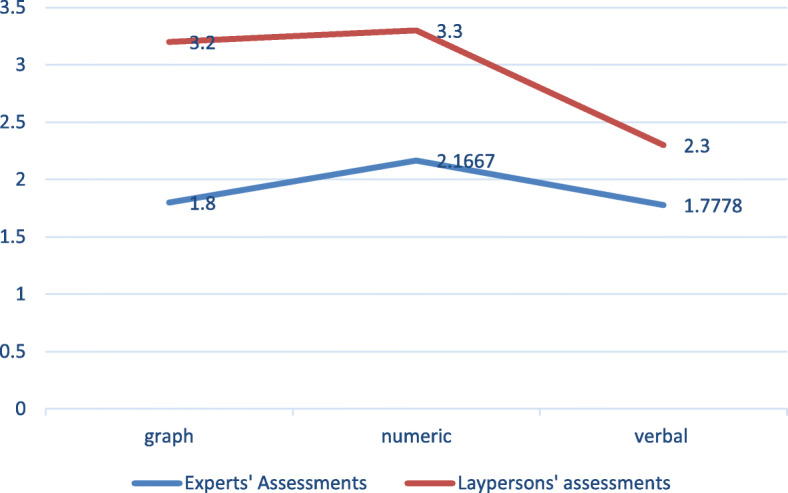
Participants’ and Experts’ assessments of gravity, for each information format

In general, both the participants and the physicians interpreted the conditions as mildly serious or not very serious. A Wilcoxon signed-rank test showed that physicians’ assessments of gravity were significantly lower than those of the laypersons (Z = − -2.828, *p* = 0.005). A follow-up Pearson chi-square test confirmed these differences (chi-square, 7.222, df = 2, *p* < 0.027). The results suggest that the participants were fairly accurate in the general trend, but tended to overestimate the conditions’ gravity in all three formats (See Fig. 4 - Participants’ and Experts’ assessments of gravity, for each information format). Looking dipper, a paired sample t-test revealed that accuracy is higher when results are explained verbally, rather than having a number stand on its own (see Table 3). Accuracy is greater when a numeric value appears in tabular form, as opposed to only a value. And overall, accuracy is greater when results are presented in a tabular form, rather than in a line graph, even though both represent deviations from the norm.

**Table 3:** Paired-sample t-test to study differences in accuracy between different displays

Health condition	Mean	Information Display	T-value	df	sig
Progesterone	0.642 1.761	verbal Information Numeric (value)	−4.897	41 45	*P* < 0.01
Progesterone	0.326 1.67	verbal information Numeric	−6.615	61 65	*P* < 0.1
Cholesterol	1.6129 1.3871	Numeric (value) Numeric (value) + History (table)	1.699	61	*P* < 0.001
Cholesterol	1.7805 1.00	Numeric (value) + History in line graph) Numeric (value) + scale + History (table)	5.193	81 81	*P* < 0.001
Infection	.7838 1.9595	Numeric (value) + scale + History (table) Numeric (value) + History in line graph	−9.866	73 73	*P* < 0.001
Infection	.7838 1.9595	Numeric (value) + History + scale (table) Numeric (value) + History in line (graph)			*P* < 0.001

Finally, we examined whether accuracy can be explained by demographic variables or participants’ general health status and familiarity with EPR use. Results of a multiple linear regression to predict level of accuracy point to a collective significant effect of gender, age, education, health status, income, EPR use, and uncertainty (F (7,201) = 24.442, *p* < .001, R2 = .460). However, only age (Beta = .186; t = − 3.063, *p* = .002), and uncertainty (Beta = −.285; t = − 5.437, *P* < 0.000) were significant predictors in the model. A one-way ANOVA revealed differences between the three age groups with regard to accuracy (F (2,208) =14.455, *p* = .000). A Tukey post-hoc test revealed that accuracy was significantly lower among those aged 18–39 (M = 0.59; std. = 0.8; *p* = 0.00) than among those aged 40–59 (M = 0.89, std. = 1.16, p = 0.00) and those aged 60+ (M = 1.26, std. = 1.26, *p* = 0.000). No significant differences were found between those aged 40–59 and those 60 years old and older. These findings suggest that age-related familiarity with different health conditions could be related to accuracy.

- H2: The three information formats (verbal, numeric, and graphic) will yield different levels of uncertainty with regards to being able to assess the condition’s level of severity.

**Fig. 5:**
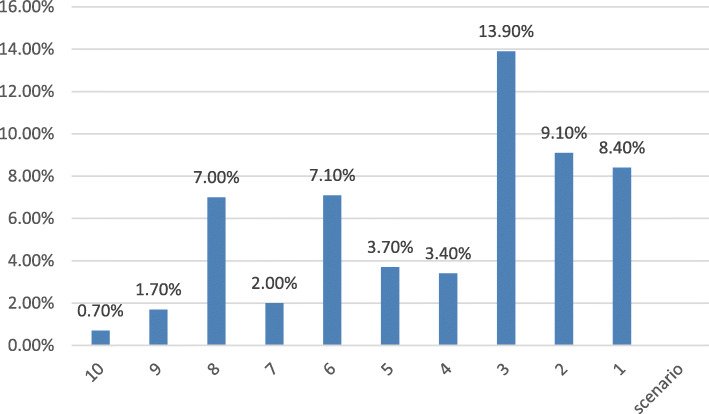
Percentage of respondents who chose the “don’t know” response in any of the 10 scenarios

We measured the proportion of respondents who chose the “don’t know” response for any of the 10 scenarios. Slightly more than half (50.7%) chose the “don’t know” option at least once. Of those, only 15% chose the “don’t know” response in more than eight scenarios. These findings indicate that the “don’t know” option was generally not chosen automatically, and without reflection. Figure 5 shows the percentage of respondents who chose the “don’t know” response in each of the 10 scenarios.

**Fig. 6:**
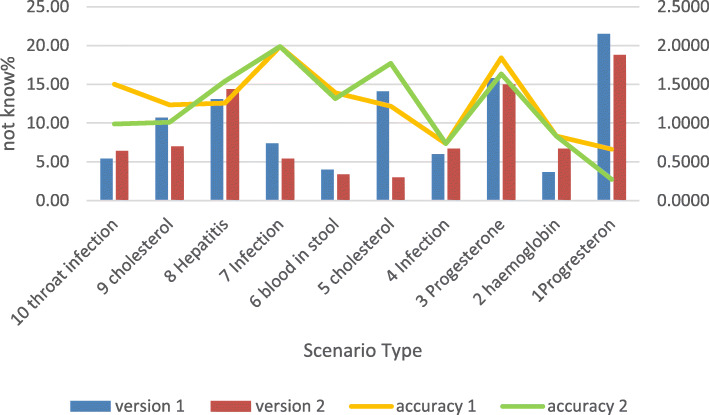
Percentage of “don’t know” answers per scenario with an accuracy trend for each version

Figure 6 demonstrates the proportion of “don’t know” responses for each scenario, and level of accuracy. The graph reveals no discernable association between either “don’t know” responses or accuracy and health condition, leading us to believe that the display of information plays an important role in both. The only visible exception relates to levels of progesterone (scenarios 1 and 3). In both scenarios the rate of “don’t know” responses is relatively high, and the level of accuracy is relatively low, compared to all other scenarios. We hypothesized that gender and age could explain these findings, and conducted two separate two-way ANOVAs to examine the effect of gender and age on accuracy and on “don’t know” responses in those two scenarios. However, no statistically significant main effects nor interaction were found.

More generally, we performed a hierarchical linear regression to predict the level of “don’t know” responses based on various demographic variables (age, gender, education, family status, and income), along with EPR use and health status. Variables were entered into the equation using the stepwise method. These variables explained a relatively small proportion of variance in uncertainty scores (“don’t know” responses). In the first model, R^2^ = .021, F (1,244) =6.214, *p* < .001. In the second model, R^2^ = .035, F (1,244) =5.92, *p* < .001. In the first model, income alone significantly predicted uncertainty scores, B = 0.512, t (244) = 6.53, *p* < .001. In the second model, both income, B = 0.512, t (244) = 6.53, *p* < .001, and gender, B = 0.512, t (244) = 6.53, *p* < .001, significantly predicted uncertainty scores. We then conducted a hierarchical linear regression to predict the level of accuracy based on demographic variables (age, gender, education, and income), along with EPR use and health status. Variables were entered into the equation using the stepwise method. In the first model, income significantly predicted accuracy, B = .228, t (205) = 3.196, R2 = .107, F (1, 205) = 2.791, *p* < .001. However, once age was entered into the equation, income was no longer significant. In the second model, age alone significantly predicted accuracy, B = .326, t (205) = 3.233, R2 = .0.92, F (1, 205) = 20.735, *p* < .001. These findings suggest that women more than men, and those of higher versus lower income, indicated that they could not assess the conditions’ gravity based on the information displayed. However, those who did were more accurate than those who were younger and of lesser means. Gender had no effect on accuracy, suggesting that women were more comfortable indicating that they were unsure of the answer than the men participating in the study.

**Fig. 7:**
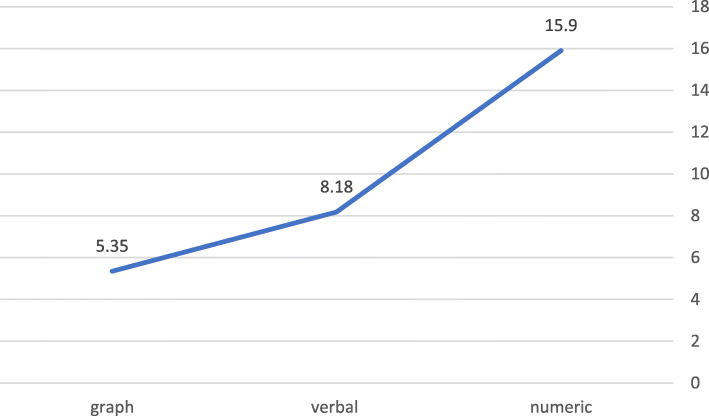
Levels of uncertainty (“don’t know”) by information format

We then performed a one-way between-subjects ANOVA to compare the effect of information format on “don’t know” responses. We found a significant effect of format type on “don’t know” responses, F (2,17) = 9.789, *p* = .001. Post hoc comparisons using the Tukey HSD test show that the mean score for the graph condition (M = 5.06, std. = 2.17) is significantly lower than that for the numeric condition (M = 15.90, std. = 4.05), and also lower than the mean score for the verbal condition (M = 9.87, std. = 5.33). On average, the numeric format produced the highest number of “don’t know” responses and the graphic format the least, indicating that respondents found the numeric format most difficult to understand and the graphic format the easiest. These findings confirmed our hypothesis that the three information formats differ in the ease with which they were understood. Yet as reported earlier, those participants who assessed the gravity of the health conditions were slightly but significantly more accurate when results appeared in a table, than in the line graph format, even though in both appeared a scale showing normal and abnormal results (see Fig. 7).

- H3: The higher the perceived gravity of the health condition, the more proactive people are likely to be in seeking help or information.

First, we performed a linear regression to predict the level of proactivity based on various demographic variables (age, sex, education, family status, having children under the age of 18, and income), and perceived gravity of the health condition. Variables were entered into the equation using the stepwise method, starting with perceived gravity and then adding the control variables one by one. Two models were found significant. In the first model, the only independent variable to predict level of proactivity was perceived gravity of the health condition, F (4,207) = 15.901, *P* < 000, *R*^*2*^ = .73. In the second model, income was found significant in addition to gravity, F (2,201) = 9.731, *P* < 001, *R*^*2*^ = .99. We conducted a one-way between-subjects ANOVA to compare the effect of information format on prefered course of action. Interestingly, we found that information format had a significant effect only on respondents’ tendency to choose “search the Internet” as a preferred course of action, F (2,16) = 3.159, *p* < .0.05. In post hoc comparisons using the LSD test, the mean score for the numeric condition (M = 62.58, std. = 8.43) was significantly higher than for the verbal condition (M = 54.8, std. = 12.69). No significant difference was found between the numeric condition and the graph condition (M = 69.71, std. = 6.55). This finding is congruent with our earlier finding that the numeric format produced the highest number of “don’t know” responses and the graphic format the least, suggesting that respondents found the numeric format hardest to understand and the graphic format the easiest. We assume that our respondents expected that searching the Internet would clarify the situation. Interestingly, however, the graphic presentation, which supposedly offers the greatest amount of contextualized information, also precipitated relatively high scores for Internet search, perhaps because of its complexity.

Finally, we expected that those who did not understand the information (“don’t know” responses) would favor more proactive measures, defined as immediately contacting their doctor or searching for information on the Internet. A Pearson correlation revealed an inverse relationship between “don’t know” responses and participants’ tendency to call a doctor (r = − 0.184, *p* < 001), and a positive relationship between “don’t know” and the other three courses of action: searching the Internet (r = 0.438, *P* < 001), waiting for the doctor to call them (r = 0.442, *P* < .005), or waiting for their next visit to the doctor (r = 0.488, *P* < .005). Thus, the less understandable the information presented, the less likely the participants were to immediately call their family doctors. Rather, they were more likely to search the Internet for information, wait for their next doctor’s appointment, or wait for their doctor to contact them. Consulting the Internet for information was positively correlated with waiting for the doctor to call and waiting for one’s next visit to the doctor (r [298] = 0.482, *P* < 0.001; r [298] = 0.467, *P* < 0.001, respectively). Thus, our hypothesis was not supported (see Table 4).

**Table 4:** Correlations between “don’t know” and preferred action (*N* = 298)

Measure	1	2	3	4
1. Call doctor Sig. (2-tailed)		−.097 .094	−.260*** .000	−.061 .296
2. Internet use Sig. (2-tailed)	−.097 .094		.482*** .000	.467*** .000
3. Wait for doctor to call Sig. (2-tailed)	−.260*** .000	.482*** .000		.779*** .000
4. Wait for visit to doctor Sig. (2-tailed)	−.061 .296	.467*** .000	.779*** .000	
5. Don’t know Sig. (2-tailed)	−.184*** .001	−.438*** .000	.442*** .000	.448*** .000

***p < 0.01

## Discussion

It has been shown that engaged patients – those who actively seek to know more about and manage their own health – are more likely than others to participate in preventive and healthy practices, self-manage their conditions, and achieve better outcomes [33]. Studies also show that engaged patients are better able to understand whether or not a result is worrisome, and what actions, if any, should be followed [34].

In our study, respondents found the numeric format hardest to understand and the graphic format the easiest. Yet they displayed a slightly higher level of inaccuracy in the graphic format, less so in the numeric, and the least in the verbal. In other words, for those respondents who hazarded a gravity assessment (as opposed to those who chose the “don’t know” option), information was most difficult to interpret correctly when presented in a line graph, and easiest to interpret correctly when presented numerically (in a tabular form). However, these differences should be explored further. Our findings concur with previous studies which suggest that graphs may appear as an appealing alternative to numbers because visualization allows for quick and intuitive assessment. However, some aspects of graph interpretation may require effortful cognitive skills that must be learned [26]. Formats that leave respondents less able to understand the results – namely, the verbal and graphic formats – produced lower inclination to actively seek professional help. Low levels of understanding (operationalized through respondents’ choice of “don’t know” when asked to assess the gravity of the information) were negatively correlated with an expectation of immediately calling the doctor, and positively correlated with searching the Internet, waiting for the doctor to contact the patient, and waiting for one’s next visit to the doctor. Thus, uncertainty regarding the meaning of the lab results drove participants to shift the burden of responsibility to their doctors, as well as to delay actively seeking medical services [23, 35]. As Zikmund-Fisher and his colleagues found in their study [13], high perceived gravity of the health condition was the only predictor of immediately calling the doctor.

Our findings suggest that age is an important predictor of both accuracy and uncertainty, indicating that familiarity with a wide range of health conditions and with the healthcare system may enhance accurate interpretation of the results. This finding is in line with the literature indicating that a broad acceptance of personal health record (PHR) technology may not be related to education or income, but to the patient’s health literacy [24, 36]. However, the participants in this study were not required to operate the EPR to elicit the test results. It is possible that low proficiency in navigating patient portals can affect older people’s effective use of these technologies.

A semantic approach to knowledge transfer posits that even if a common syntax or language is present, differences of interpretation can impede communication between experts and laypersons [37–40]. As suggested by Witteman and Zikmund-Fisher [34], patients viewing laboratory results may not care about the number itself. Instead, they wish to know: “Is this good or bad?” or, more personally, “Am I OK?” or “Do I need to do anything?” ([34], p. 360). Moreover, simply understanding the plain meaning of medical information may not be enough to interpret the information’s significance. For instance, it is rarely enough to understand the meaning of each isolated result, to truly assess the gravity of a health condition, patients need to grasp the comprehensive meaning of the results. To move patients from adherence to engagement, personalized information must be presented in a way that ensures precision of interpretation, not only informing patients, but allowing them to act on the information [41]. And so, to engage individuals in their health, it is critical that the numbers, values, terms, and units have meaning for the person receiving them, and can easily become actionable.

A well-designed results sheet can and should encourage patients to take an active role in interpreting their test results in ways that will allow them to follow up on their health. We suggest that graphs, tables, and charts could be made easier to interpret if coupled with a brief and concise verbal explanation, using language that is familiar to readers. Moreover, instead of indicating a diversion from the norm, it might be more helpful to indicate an overall level of urgency, and include a recommendation for the type of follow-up required (i.e., a consultation with a doctor, more tests, or certain types of monitoring).

### Limitations

Our findings should be considered in light of the study’s limitations. First, though the information presented was drawn from authentic records, our respondents encountered it in the context of hypothetical scenarios. This method has been documented in the literature on EPR design (e.g., [13]). Nonetheless, addressing hypothetical scenarios meant that the participants lacked the personal relevance that such test results have for patients attempting to manage these conditions. Relatedly, we did not measure respondents’ familiarity with the specific tests presented. We assumed that many of our respondents were familiar with at least some of the conditions in the scenarios, and had perhaps even managed them in the past. We also assumed that there are many scenarios in which patients with no prior knowledge of a given laboratory test might view laboratory results in a patient portal. We therefore believe that our study design successfully simulated realistic circumstances. However, it is possible that our results may not accurately reflect how people respond to their own personalized health information. Future inquiries should consider further how people might interpret their own health information, using a combination of quantitative and qualitative methods.

Second, the sample size, and level of attrition in studies such as this need be addressed in further studies. It is possible that the length of the questionnaire and its complexity contributed to both. Furthermore, despite our sample’s diversity in terms of demographics, experience with the EPR system and health beliefs, our research design prevented us from reaching minority groups within the Israeli population, such as Arabs, ultraorthodox Jews, and immigrants from the former Soviet Union or of Ethiopian or French descent. Their omission from the study was partly due to methodological complications. In particular, the language used in the EPR is Hebrew, but for many Israeli minorities Hebrew is a second language. Asking minority respondents to fill in the questionnaire in Hebrew would have required a control for language proficiency that was beyond the scope of this study; while the alternative, translating the scenarios into Russian, Amharic, Arabic or French, might have reduced the authenticity of the information. In the present case, we can assume that if native Israelis, highly proficient in Hebrew, demonstrated significant deficiencies in their comprehension of personalized medical information, members of these populations would do so as well. However, we encourage scholars to study the role of cultural and educational diversity in the interpretation of personalized health information. To further our understanding on how information presentation affects laypersons’ understanding, perceptions and actions, future studies should design methodologies that can survey larger and more diversified populations.

## Conclusion

To conclude, this study makes three unique contributions. First, it is concerned not only with assessments of urgency, but with the accuracy of patients’ assessments. Second, it uncovers how people react when they are unsure what the results encountered in electronic records mean. Third, it examines which follow-up actions laypersons are likely to take in response to their interpretation of the results. As such, the paper deals with the core problem of digitation – namely, how to make medical information understandable so that it can be translated into appropriate and timely action. Addressing these concerns is key to designing health information technologies that can improve laypersons’ engagement and self-care, as well as reduce both under- and over-utilization of health services.

## Data Availability

The datasets generated during and/or analysed during the current study are not publicly available due to the originality of the survey questions, but are available from the corresponding author on reasonable request.

## Abbreviations

RPC: Remote Patient Care
EPR: Electronic Patient Records
HIT: Health information technologies
NIS: New Israeli Shekels
HMO: Health Maintenance Organizations
